# VEGF_121_b and VEGF_165_b are weakly angiogenic isoforms of VEGF-A

**DOI:** 10.1186/1476-4598-9-320

**Published:** 2010-12-31

**Authors:** Raúl Catena, Leyre Larzabal, Marta Larrayoz, Eva Molina, Jose Hermida, Jackeline Agorreta, Ramon Montes, Ruben Pio, Luis M Montuenga, Alfonso Calvo

**Affiliations:** 1 Laboratory of Novel Therapeutic Targets. Division of Oncology, Center for Applied Medical Research (CIMA). University of Navarra, Pamplona, Spain; 2 Laboratory of Biomarkers. Division of Oncology, Center for Applied Medical Research (CIMA). University of Navarra, Pamplona, Spain; 3 Division of Cardiovascular Sciences, Laboratory of Thrombosis and Haemostasis, Center for Applied Medical Research. University of Navarra, Pamplona, Spain

## Abstract

**Background:**

Different isoforms of VEGF-A (mainly VEGF_121_, VEGF_165 _and VEGF_189_) have been shown to display particular angiogenic properties in the generation of a functional tumor vasculature. Recently, a novel class of VEGF-A isoforms, designated as VEGF_xxx_b, generated through alternative splicing, have been described. Previous studies have suggested that these isoforms may inhibit angiogenesis. In the present work we have produced recombinant VEGF_121/165_b proteins in the yeast *Pichia pastoris *and constructed vectors to overexpress these isoforms and assess their angiogenic potential.

**Results:**

Recombinant VEGF_121/165_b proteins generated either in yeasts or mammalian cells activated VEGFR2 and its downstream effector ERK1/2, although to a lesser extent than VEGF_165_. Furthermore, treatment of endothelial cells with VEGF_121/165_b increased cell proliferation compared to untreated cells, although such stimulation was lower than that induced by VEGF_165_. Moreover, *in vivo *angiogenesis assays confirmed angiogenesis stimulation by VEGF_121/165_b isoforms. A549 and PC-3 cells overexpressing VEGF_121_b or VEGF_165_b (or carrying the PCDNA3.1 empty vector, as control) and xenotransplanted into nude mice showed increased tumor volume and angiogenesis compared to controls. To assess whether the VEGF_xxx_b isoforms are differentially expressed in tumors compared to healthy tissues, immunohistochemical analysis was conducted on a breast cancer tissue microarray. A significant increase (p < 0.05) in both VEGF_xxx_b and total VEGF-A protein expression in infiltrating ductal carcinomas compared to normal breasts was observed. A positive significant correlation (r = 0.404, p = 0.033) between VEGF_xxx_b and total VEGF-A was found.

**Conclusions:**

Our results demonstrate that VEGF_121/165_b are not anti-angiogenic, but weakly angiogenic isoforms of VEGF-A. In addition, VEGF_xxx_b isoforms are up-regulated in breast cancer in comparison with non malignant breast tissues. These results are to be taken into account when considering a possible use of VEGF_121/165_b-based therapies in patients.

## Background

Angiogenesis is a process by which new blood vessels are formed from pre-existing ones [1]. In physiological conditions, this process is strictly controlled by a set of molecules that can either activate the process (proangiogenic factors) or inhibit it (antiangiogenic factors) [2]. During the last decades, it has been widely established that solid tumors have abnormal hyperactivation of angiogenesis [2]. Among the factors that can trigger angiogenesis, the lack of oxygen (hypoxia) is of special importance. Virtually all solid tumors eventually activate angiogenesis in order to overcome lack of oxygen and nutrients after reaching a certain burden [3,4]. One of the most important mediators of hypoxia-activated angiogenesis is the Vascular Endothelial Growth Factor (VEGF-A), produced by tumor cells after sensing low oxygen levels [5,6]. VEGF-A expression can also be induced by non-hypoxia mediated activation, such as Ras signalling [7].

VEGF-A is a key player in tumor-induced angiogenesis, and its overexpression has been found in most solid tumor types [6]. VEGF-A acts through its cognate receptors VEGFR1 (Flt-1) and VEGFR2 (Flk-1/KDR), in endothelial and bone marrow-derived cells [6,8]. The VEGF pathway has been used as a major target to block tumor angiogenesis. A set of molecules that bind and inhibit different components of the VEGF-A pathway have been developed during the past years. Some of them have already reached the clinical practice, such as bevacizumab (Avastin^®^, Genentech), a monoclonal antibody that binds and inactivates VEGF-A, or sunitinib (Sutent^®^, Pfizer), a tyrosine-kinase inhibitor that blocks phosphorylation of several tyrosine-kinase receptors including VEGFR1 and VEGFR2 [9,10].

The VEGF-A gene contains 8 exons, which can give rise to 5 main alternatively spliced isoforms (VEGF_121_, VEGF_145_, VEGF_165_, VEGF_189 _and VEGF_206_) [6]. Alternative translation codons upstream of the canonical ATG codon can be used, so that longer isoforms can also be generated [11]. However, the relative importance of these members is still undetermined. Recently, a novel set of isoforms, the so-called "b-isoforms" or "VEGF_xxx_b" isoforms, have been described. These transcripts of the VEGF-A gene code for polypeptides with the same length as the classical ones, because exon 8 (present in all the formerly known isoforms) is substituted by an alternatively spliced exon of the same size (exon 8b) [12]. These isoforms were therefore named VEGF_121_b, VEGF_165_b, VEGF_189_b etc. In the classically studied isoforms, exon 8 is known to be important for receptor activation [13]. Thus, the "b-isoforms", where exon 8 is substituted by another peptide sequence, were hypothesized to act as potential antagonists of VEGF-A receptors [14]. Several reports have indeed shown that VEGF_165_b may have anti-angiogenic properties [14,15], while others cast some doubts about such activities, suggesting that it may act as a VEGF-A receptor agonist [16,17].

Another interesting issue is the possible differential expression between "angiogenic" vs. "antiangiogenic" isoforms in pathologies where development of aberrant vasculature is involved, including cancer. Previous studies have shown in a limited number of samples, using semiquantitative RT-PCR, that VEGF_xxx_b isoforms are highly expressed in normal prostate, colon and kidney compared to their malignant counterparts [14,18,19]. It was proposed that formation of neovasculature in pathological conditions would modify alternative splicing of VEGF-A, thus promoting the expression of the "b-isoforms" (supposedly anti-angiogenic) at the expense of the classical angiogenic family of isoforms. This would also be extremely interesting because expression of the ratio VEGF_xxx_b/VEGF could be utilized as a biomarker of angiogenic disease [13].

Since a therapeutic approach using recombinant VEGF_xxx_b proteins is very attractive, but the biological activity of such transcripts is not yet clear, we sought to produce recombinant VEGF_121_b and VEGF_165_b proteins in the yeast *Pichia pastoris*, and constructed expression vectors to overexpress these isoforms, in order to further elucidate their role in cancer models. In addition, we analyzed protein expression of VEGF_xxx_b and total VEGF in normal mammary glands and 50 breast cancer samples, using specific antibodies previously characterized.

## Methods

### Cloning of VEGF_121_b and VEGF_165_b

Oligonucleotides were purchased to Sigma-GenoSys (Sigma, St. Louis, MO, USA). The primers VF (5'GAAACCATGAACTTTCTGCTGTCTT3') and V121bR (5' TT**AAGCTT**TCAGTCTTTCCTGGTGAGAGATTTTTCTTGTCTTGCTCTATC3') were used to clone the VEGF_121_b isoform by PCR into pCR2.1 vector (Invitrogen). VF and V165bR (5' TT**AAGCTT**TCAGTCTTTCCTGGTGAGAGATCTGCAAGTAC

GTTCGTTTAACTC 3') were used to clone VEGF_165_b. Note that initiation codon is underlined in VF and both reverse oligonucleotides contain H*ind*III restriction sites (bold). VEGF_121_b and VEGF_165_b coding sequences were then subcloned into the pCDNA3.1(-)Neo expression plasmid. The primer VPPF (5' GGTCTCGAGAAAAGAGAGGCTGAAGCTGCACCCATGGCAGAAGG 3'), together with V121bR or V165bR, was used to clone VEGF_121_b and VEGF_165_b coding sequences lacking the signal peptide (ΔPSVEGF_xxx_b) into the pPICZalphaC vector (Invitrogen) for production of recombinant proteins in the yeast *Pichia pastoris*, where the alpha-factor signal peptide is used to achieve extracellular expression of the VEGF_xxx_b sequences.

### Recombinant protein production and purification

The pPICZalphaC plasmids carrying the ΔPS-VEGF_xxx_b sequences were linearized, gel-purified, and measured for concentration. 80 μL of *Pichia pastoris *cells were mixed with 5 μg of linearized-plasmid in 1 mm-wide electroporation cuvettes (Bio-Rad). Electroporation was carried out in a Gene-pulser II (Bio-Rad) using the preset yeast conditions. After electroporation, 1 mL of 1 M sorbitol was added to the cuvettes and the electroporated cells were transferred to sterile microtubes. Yeasts were incubated at 30°C for 2 h and then spread in YPDSZ plates (1% yeast extract, 2% peptone, 2% sorbitol, 2% agar and 100 μg/mL zeocin) and incubated for 9 days at 29°C. Zeocin-resistant colonies were picked and grown in YPD medium. Yeast clones were transferred to BMGY medium (1% yeast extract, 2% peptone, 100 mM potassium phosphate pH 6.0, 1.34% yeast nitrogen base, 0.00004% Biotin, 1 U/mL gentamycin sulphate, and 1% glycerol) to allow cells to grow exponentially for 30 h at 29°C and thorough shaking. Yeasts were then centrifuged and resuspended in BMMY medium (the same composition as BMGY but containing 1% methanol instead of glycerol) to induce protein production. Supernatants were collected 24 hours after incubation at 29°C and thorough shaking, and analyzed by SDS-PAGE to determine the best clone producing each of the VEGF_121/165_b isoforms. Selected clones were grown at 29°C in 2 L of BMGY for 2 days and then changed to BMMY inducing medium, in order to produce large amounts of recombinant products. For purification, nickel-affinity chromatography was used. A Hi-Trap chelating column (Amersham) was connected to an AKTÄ High Pressure Liquid Chromatography (HPLC) device (Amersham). *Pichia pastoris *supernatants containing recombinant VEGF_121/165_b proteins were diluted in binding buffer (0.02 M sodium phosphate, 1 M NaCl, pH 7.2; all reagents from Sigma) and loaded into the HPLC device. Elution buffer (0.02 M sodium phosphate, 1 M NH_4_Cl, 500 mM Imidazole, pH 7.2; all reagents from Sigma, except for imidazole, purchased from Merck) was loaded, and gradually mixed with binding buffer with an increasing proportion of elution buffer. Fractions of 1 mL were collected throughout the process.

Purified proteins after affinity chromatography were depleted from eluting medium and changed to PBS through dialysis, using Slide-A-lyzer cassettes (Pierce) with a 10 KDa threshold pore. Cassettes were filled with eluted protein and drawn in 3L of PBS, overnight. This step was repeated with new PBS for 6 more hours. Dialyzed proteins were extracted from the cassettes with syringes and snap frozen.

### Protein extraction, electrophoresis and western blot

For protein extraction, cultured cells were lysed for 30 min at 4°C in RIPA buffer (50 mM Tris pH 7.4, 150 mM NaCl, 1 mM PMSF, 1 mM EDTA, 1% sodium deoxycholate and 0.1% sodium dodecyl sulphate; all reagents from Sigma) plus a protease inhibitor cocktail (Roche, Switzerland). Samples were then centrifuged at 13000 rpm. Protein concentration was determined by the bicinchoninic acid protein assay (Pierce, Rockford, IL). In the case of conditioned culture media, supernatants were centrifuged to get rid of any cell debris and 20-fold concentrated by centrifugation for 45 min, using 15-KDa Amicon Ultra centricons (Millipore, Billerica, MA, USA).

Proteins were electrophoresed in Bis-Tris buffered gels (Novex gels, Invitrogen) in either reducing or non-reducing conditions, following standard procedures. 20 μg protein solution (in RIPA buffer) were mixed with Laemmli sample buffer and boiled for 5 min. Electrophoresis was carried out in 1X running buffer for 90 min at 130V and room temperature. Proteins were directly stained in the gel with Coomassie blue or transferred to PVDF membranes for immunodetection. Deglycosylation analysis of VEGF_xxx_b proteins (90 μM) was treated with 0.8 mM Endo F1 and incubated for 1 h at 37°C; cleavage was monitored by SDS-PAGE.

Membranes for western blots were rinsed twice with PBS-tween, blocked with PBST plus 5% skim milk for 30 min at room temperature, and incubated with primary antibodies. Antibodies against VEGF (MAB293, R&D; and sc-152, Santa Cruz), VEGF_xxx_b (MAB3045, R&D), pKDR, total KDR, pERK1/2, total ERK1/2, and GAPDH (all of these latter ones from Cell Signalling) were used. Then, horseradish peroxidase-labelled secondary antibodies (GE Healthcare) against the corresponding primary antibodies were added. Immunoreactive bands were visualized by a chemoluminescent method using the Lumi-lightPLUS kit (Roche).

### Cell Culture

HUVECs, PC3 and A549 cell lines were obtained from the American Type Culture Collection (ATCC, Manassas, VA, USA). PC3 and A549 cells were maintained in complete medium, consisting of: RPMI-1640 growth medium (Invitrogen) with Glutamax^®^, supplemented with 10% heat-inactivated FBS, 100 U/mL penicillin and 100 μg/mL streptomycin (both antibiotics from Invitrogen). HUVECs were maintained in EGM-MV2 medium (Lonza) containing human recombinant EGF, VEGF, FGF, IGF-1, hydrocortisone, ascorbic acid and 2% FBS. Cell culture medium with 1% serum was used to analyze cells supernatants by western blot.

Purified plasmidic DNA was introduced into mammalian cells through cationic lipid-based transfection with the reagent Lipofectamine 2000, according to manufacturer's recommendations. Transfected cells were selected and maintained with complete medium plus 300 μg/mL (PC3) or 500 μg/mL (A549) G418.

### Cell proliferation assays

Two different methods were used to assess cell proliferation. The first method consisted of the MTT (Sigma-Aldrich, Italy) assay. Cells were seeded in 50 μL 2% FBS-containing growth medium in 96-well culture plates and allowed to attach overnight. Two-fold concentrated recombinant human VEGF_165 _(rhVEGF_165_, Apollo Cytokine Research), recombinant human VEGF_121_b and VEGF_165_b produced in *Pichia pastoris *(VEGF_121_b(pp) and VEGF_165_b(pp)), recombinant human VEGF_165_b produced in CHO cells (VEGF_165_b(hs)), kindly provided by Dr. David O. Bates (Microvascular Research Laboratories, Department of Physiology, University of Bristol, UK), or the VEGFRs inhibitor GW654652 (GlaxoSmithKline) were added. In each time point, 10 μL 5% MTT solution was added to each well. Plates were incubated for additional 3 h at 37°C. The resulting formazan crystals were finally solubilized by administration of 100 μl 10% SDS in 50% N-N-Dimethylformamide to each microplate well. Absorbance at 550 nm was measured using a TECAN Sunrise microplate reader. Wells containing only complete medium were used as controls. Each experiment was performed three times using six replicates for each drug concentration.

The second method consisted of analysis of DNA synthesis by incorporation of the modified nucleotide EdU, using the Click-it reaction according to the manufacture's instructions (Invitrogen). Briefly, cells were plated at 50% confluence and treated with 50 or 100 ng/mL of rhVEGF_165_, VEGF_121_b(pp), VEGF_165_b(pp), or bFGF overnight. Cells were then incubated for 1 h with 5 mM EdU solution, washed, trypsinized, fixed, permeabilized, and incubated with Alexa-Fluor-647 dye in the presence of copper for catalysis of the Click-it reaction. Cells were analyzed with a FACScalibur flow cytometer to determine EdU incorporation.

### Tumor xenograft and Matrigel plug assays

*Nu/Nu *mutant athymic mice (Balb/C genetic background) were purchased from Harlan Laboratories (Barcelona, Spain) and maintained in SPF (Specific Pathogen Free) standard conditions. One million PC3 or five million A549 cells and their corresponding transfectants in exponential growth phase were resuspended in 200 μL PBS and injected subcutaneously in the flanks of *Nu/Nu *mice. Tumor measurements were done with precision callipers and animals were sacrificed before tumors reached 1.7 cm in diameter. Experiments were conducted according to the guidelines for ethical use of animals of our Institution (CIMA-University of Navarra) under an approved protocol. Tumors were harvested and fixed overnight in 10% buffered formalin, embedded in paraffin, and sectioned. Primary tumor volumes were calculated with the formula: V = length × (width)^2^/2.

For Matrigel plug assays, 400 μL Growth Factor Reduced Matrigel (BD) were mixed with 100 ng of rhVEGF_165_, VEGF_121_b(pp), VEGF_165_b(pp), or bFGF (as positive control) in 100 μL PBS and injected subcutaneously in *Nu/Nu *mice. One week after cell inoculation, mice were injected retro-orbitally with 100 mL Fluorescein-labelled dextran (3 mg/mL) or with Alexa-647-labelled isolectin B4 (100 μg/mL). After 15 min, mice were sacrifized, and the Matrigel plugs were explanted and analyzed under a Zeiss Axiovert confocal microscope.

### Immunohistochemistry, fluorescence analysis and quantification

Tissues (xenografted tumors or matrigel plugs) were obtained from the *in vivo *experiments, fixed in 10% buffered formalin and embedded in paraffin. Tissue Microarray (TMA) slides were obtained from AccuMax (Seoul, Korea; catalogue # A202(I)). This TMA contains 100 breast tissue cores from 50 patients and 8 tissue cores from 4 normal breast tissue obtained by mammoplasty. Breast cancer types include 33 infiltrating ductal carcinomas, 7 papillary carcinomas, 3 phyllodes tumors, 4 infiltrating lobular carcinomas, and 3 samples corresponding to other breast cancer tumor types.

For immunohistochemistry, slides were deparaffinized, hydrated, and incubated for 10 min with 3% H_2_O_2 _in water to quench the endogenous peroxidase activity. An antigen retrieval method was used for detection of the antibodies. Dilutions of primary antibodies were as follows: 1:200 for Caspase 3 (Cleaved Caspase-3 Asp 175, Cell Signaling); 1:20 for CD-31 (Dianova); 1:100 for PDGFRβ (Cell Signaling); 1:200 for VEGF (Santa Cruz); 1:50 for VEGF_xxx_b (R&D). Primary antibodies were incubated at 4°C overnight or for 1 h at RT in the case of CD31. Tissues were washed in TBS and incubated with the appropriate secondary antibody. Afterwards, slides were incubated for 30 min with the EnVision™ anti-rabbit detection system (Dako). Peroxidase activity was carried out with DAB (Dako). Finally, slides were counterstained with hematoxylin, dehydrated, and mounted. For quantifications of immunohistochemistry in xenografted tumor sections, 10 random images (200×) per mouse were captured with a microscope (Leica, Wetzlar, Germany) equipped with the Analysis™ software. Positive cells were quantified with Image J (NIH, Bethesda, MD, USA). Measurements are given as relative area occupied by the stained tissue with respect to the reference area (for CD-31 and PDGFRβ) or percentage of positive cells (for active caspase-3). For the TMA, a semiquantification procedure was performed according to a score that takes into account the extension and intensity of the immunoreactivity signal. The "0" score was assigned when the sample was completely negative. Score "1" meant weak signal; Score "2", strong and widespread signal; and Score "3", very strong and widespread signal. Signal was considered positive when at least 10% of the cells were positive.

For quantification of FITC-dextran and Alexa-647 Isolectin B4 in sections of Matrigel plugs, slides were analyzed with an Axiovert (Carl Zeiss, Germany) epifluorescence microscope and 10 random pictures of each Matrigel were taken. Labelled area was measured with the ImageJ software.

### Statistical analysis of data

Data sets were tested for normal distribution with Shapiro-Wilks and Kolgomorov-Smirnoff tests. Levene's test was also performed to verify homogeneity of variances. In those cases where tests displayed normal distribution, ANOVA was performed to test for possible differences among groups. Bonferroni correction was used for post-hoc comparison in the case of variance homogeneity, whereas Tamhane's correction was the choice in the case of Levene's positive test. For non-normal distributed data sets, non-parametric tests were applied: Kruskal-Wallis for multiple comparisons and Mann-Whitney's U-test with significance correction for double comparisons of independent samples. Wilcoxon's test was carried out for dependent sample data. To run these tests, The SPSS software was used. Results with a p-value < 0.05 were considered significant (*), and those with a p-value < 0.01 (**), or <0.001 (***), very significant.

## Results

### Generation of *Pichia pastoris*-derived clones with VEGF_121/165_b overexpression and purification of recombinant proteins

**Figure 1:**
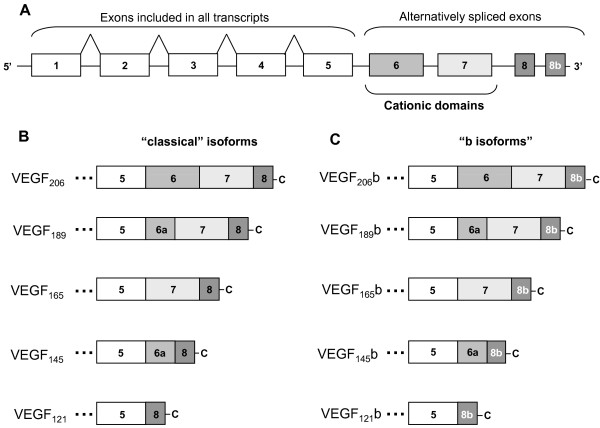
**VEGF-A transcripts generated by mRNA alternative splicing**. Exon organization of VEGF-A gene (A). Exons 1 to 5 are included in all isoforms (A-C). The 3' alternative exons 8 and 8b, of the same length in base pairs, are differentially included in the "classical" family of VEGF-A isoforms (B) or the novel family of "b isoforms" (VEGF_xxx_b) (C).

**Figure 2:**
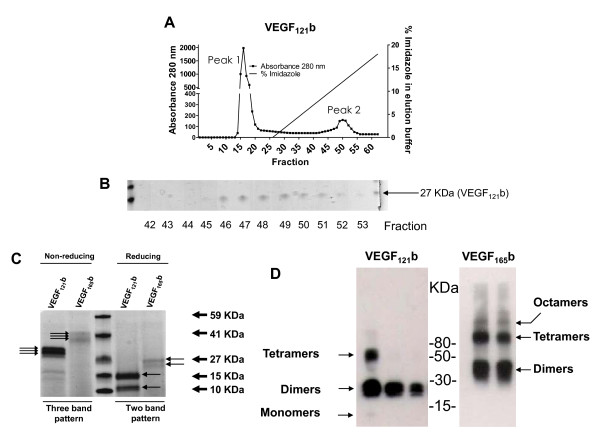
**Purification of recombinant VEGF_xxx_b produced in *Pichia pastoris***. HPLC elution profile showing the absorbance overtime, after production of VEGF_121_b (A). Two peaks are observed for both isoforms: Peak 1 corresponds to proteins in the *P. pastoris *culture supernatants unbound to the column, whereas peak 2 corresponds to the eluted VEGF_121_b protein. Fractions indicated in the graphs were electrophoresed and stained with Coomassie blue. Bands of the expected size were observed in the fractions corresponding to peak 2 (B). Culture supernatants subjected to electrophoresis for both VEGF_165_b and VEGF_121_b. Under non-reducing conditions the 3-band pattern (light arrows) corresponds to dimers (most likely glycosylated-glycosylated, glycosylated-non-glycosylated and non-glycosylated-non-glycosylated proteins, as previously described for the VEGF-A classic isoforms). Under reducing conditions, bands (2-band pattern, as described for VEGF-A under these conditions) correspond to monomers (C). VEGF_121/165_b proteins purified after production in the yeast *P. pastoris *are strongly immunoreactive with an antibody raised against VEGF_xxx_b. The lanes in the blots show bands of 3 clones with different amounts of secreted VEGF_121_b, or 2 clones in the case of VEGF_165_b (D).

Figure 1 shows a scheme of the different exons included in each of the "classical" VEGF-A isoforms and the "VEGF_xxx_b" isoforms. The coding sequence for both VEGF_121_b and VEGF_165_b, lacking the native human signal peptide, was cloned into the pPICZalphaC plasmid. The native human signal peptide sequence was substituted by the yeast alpha-factor signal peptide in these constructs, which is known to be very efficient for secretion in *Pichia pastoris*. To obtain top-quality recombinant proteins, VEGF_xxx_b was purified from *Pichia pastoris *culture supernatants through nickel-affinity chromatography. Figure 2A shows a chromatographic image of the procedure. Recombinant VEGF_121_b was eluted in a gradient of 20% imidazole. Peak 1 corresponds to all unbound substances, which run through the column. Peak 2 corresponds to proteins eluted with approximately 12.5% of imidazole (Figure 2A). Similar results were found for VEGF_165_b (not shown). Figure 2B shows Coomasie blue staining of different aliquots taken during elution of VEGF_121_b around peak 2, where the bands correspond to the expected size of VEGF_121_b. These results show that nickel-affinity is an efficient method to purify VEGF_xxx_b proteins from *Pichia pastoris *culture supernatants.

Electrophoresed culture media from *Pichia pastoris *clones obtained after electroporation with the linearized VEGF_121_b or VEGF_165_b sequence containing pPICZaphaC plasmids and selection with zeocin for one week is shown in Figure 2C. Bands of the expected molecular masses were detected in supernatants from *Pichia pastoris *clones. The amount of ectopic protein was clearly visible among the total secreted proteins by this yeast. The clones overexpressing higher amounts of recombinant VEGF_xxx_b proteins were chosen for large-scale production. Importantly, *Pichia pastoris*-derived recombinant proteins were immunoreactive to the currently available (and previously validated) commercial antibody (R&D) against VEGF_xxx_b (Figure 2D).

### Recombinant VEGF_121/165_b isoforms expressed in *Pichia pastoris *form dimers

The band pattern of recombinant human VEGF_121/165_b isoforms expressed in *Pichia pastoris *was similar to that of the native VEGF_xxx _isoforms, as previously described [20]. VEGF_121_b forms dimers that can be detected in the gel as three bands under non-reducing conditions (Figure 2C). The three bands probably correspond to dimers of glycosylated-glycosylated, glycosylated-non-glycosylated and non-glycosylated-non-glycosylated proteins, as described for the VEGF-A classic isoforms [20]. The same culture supernatants run under reducing conditions showed only two bands (Figure 2C), which would be concordant with the ability of these proteins to dimerize. The same pattern can be seen for VEGF_165_b (Figure 2C), although the bands are not as clearly seen as for VEGF_121_b. Recombinant VEGF_xxx_b proteins also formed larger complexes, such as tetramers and octamers, especially in the case of VEGF_165_b (Figure 2D).

To assess the glycosylation status of the VEGF_xxx_b recombinant proteins produced in *Picha pastoris*, endoglycosidase F1 was used both in reducing and non-reducing conditions (Additional file 1 Figure S1). VEGF_121/165_b deglycosylation produced an electrophoretic shift for both isoforms. Molecular weights for both glycosylated and deglycosylated proteins are compatible with those observed for VEGF_121 _and VEGF_165 _[20,21].

### Recombinant VEGF_121/165_b proteins induce proliferation of HUVEC cells

**Figure 3:**
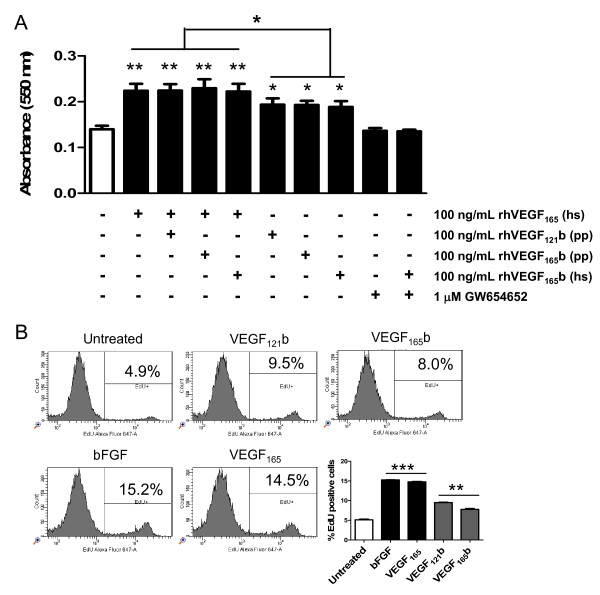
**VEGF_121/165_b isoforms induce proliferation of endothelial cells in culture through VEGFRs activation, although to a lesser extent than VEGF_165_**. **A: **MTT assays. Commercial recombinant VEGF_165 _(R&D systems), VEGF_121_b and VEGF_165_b isoforms produced in *P. pastoris *(pp), and VEGF_165_b produced in mammalian cells (hs) were added to HUVECs alone or in combination. VEGF_165 _induces proliferation (p < 0.01) of HUVECs by 63% compared to control untreated cells. Co-administration of VEGF_121_b (pp), VEGF_165_b (pp) or VEGF_165_b (hs) with VEGF_165 _does not abrogate this effect. Addition of VEGF_121/165_b isoforms alone induces proliferation of HUVECs by ~40% over untreated cells. Co-treatment with VEGF_165_b (hs) and the VEGFR inhibitor GW654652 (1 μM) blocks the effect on proliferation. **B: **Analysis of DNA synthesis by incorporation of the modified nucleotide EdU. Administration of bFGF and VEGF_165 _increases DNA incorporation into HUVECs by 3-fold compared to untreated controls. Exposure to VEGF_165_b(pp) and VEGF_121_b(pp) also increases significantly DNA incorporation (by almost 2-fold) as compared to controls. *: p < 0.05; **: p < 0.01; ***: p < 0.001.

The effect of VEGF_121/165_b recombinant proteins produced in our laboratory (VEGF_121_b(pp) and VEGF_165_b(pp)) was first tested on endothelial cell proliferation *in vitro*. We also tested the effect of a VEGF_165_b recombinant protein (VEGF_165_b(hs)) produced in mammalian CHO cells to discard any possible yeast-glycosylation-derived effect. A first experiment was conducted with the MTT assay. Addition of 100 ng/mL commercial VEGF_165 _(from R&D) induced HUVEC proliferation by 63% compared to untreated cells (Figure 3A). Co-administration of VEGF_165 _and either VEGF_121_b(pp), VEGF_165_b(pp), or VEGF_165_b(hs) (at the same dose) caused a similar proliferative induction (Figure 3A). Exposure of HUVECs to each one of the recombinant "b-isoforms" alone resulted in ~40% increased proliferation. Administration of the VEGFR-targeting compound GW654652 alone or with VEGF_165_b(hs) produced similar rates of HUVECs proliferation than untreated control cells (Figure 3A). This result shows the specificity of VEGF_165_b in inducing VEGFR-mediated endothelial proliferation. Similar results using the VEGFR inhibitor were obtained for VEGF_121_b(pp) and VEGF_165_b(pp) (results not shown).

To confirm our results on proliferation we used an alternative method, based on DNA incorporation into the cells. As shown in Figure 3B, administration of bFGF and VEGF_165 _increased DNA incorporation into HUVECs by 3-fold (p < 0.001), compared to untreated controls. Exposure to VEGF_165_b(pp) and VEGF_121_b(pp) also increased significantly (p < 0.01) DNA incorporation into HUVECs by almost 2-fold as compared to controls. Therefore, these experiments parallel those obtained by MTT showing that VEGF_xxx_b isoforms increase proliferation, although less potently than VEGF_165_.

### VEGF_121/165_b induces phosphorylation of Flk-1/KDR and its downstream effector p42-44/ERK

**Figure 4:**
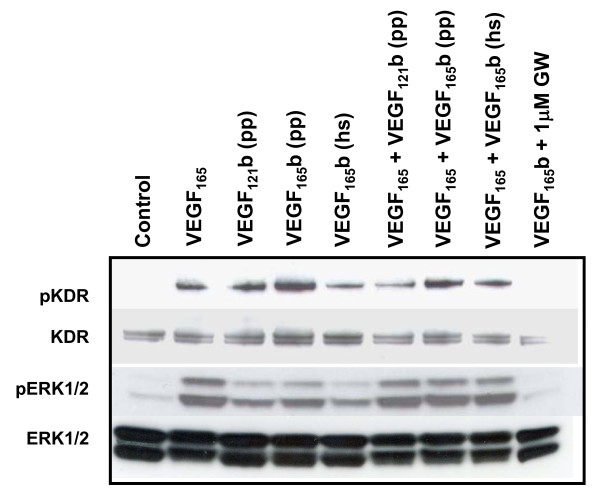
**VEGF_121/165_b isoforms activate the ERK signalling pathway through VEGF receptors**. Effect of VEGF_121/165_b isoforms on downstream signalling pathways, using antibodies against Y1175 in VEGFR2 (KDR), S473 in AKT and T202/Y204 in ERK1/2, after treatment of HUVECs with 100 ng/mL of each cytokine. VEGF_165 _stimulates phosphorylation of VEGFR2 and ERK1/2. This effect is not blocked by co-administration of either VEGF_121_b from *P. pastoris *or VEGF_165_b of different origins (mammalian cells or *P. pastoris*). Furthermore, addition of VEGF_121/165_b isoforms alone produces signalling activation as well. KDR/ERK1/2 phosphorylation was shown to be mediated specifically through VEGF receptors, since treatment with 1 μM of the specific inhibitor GW654652 blocks phosphorylation of the receptor and the intracellular transducer.

Addition of all VEGF-A proteins to HUVECs induced phosphorylation of the VEGF-A receptor Flk-1/KDR (also known as VEGFR2) and the intracellular kinase ERK1/2 (Figure 4). As expected, VEGF_165 _caused phosphorylation of KDR and ERK1/2, 10 min after treatment in serum-free conditions (Figure 4). VEGF_121/165_b proteins induced KDR phosphorylation in HUVECs as well. VEGF_121_b and VEGF_165_b, either produced in mammalian cells or in *Pichia pastoris *stimulated similarly ERK1/2 phosphorylation. Co-administration of VEGF_165 _and VEGF_121/165_b did not prevent VEGF_165_-induced KDR or ERK1/2 phosphorylation. VEGF_165_b(pp) failed to activate the KDR-ERK pathway in the presence of the VEGFRs inhibitor GW654652, thus indicating receptor specificity. Similar results were obtained with VEGF_121_b(pp) and VEGF_165_b (hs) (not shown).

### VEGF_121/165_b isoforms stimulate angiogenesis *in vivo*

**Figure 5:**
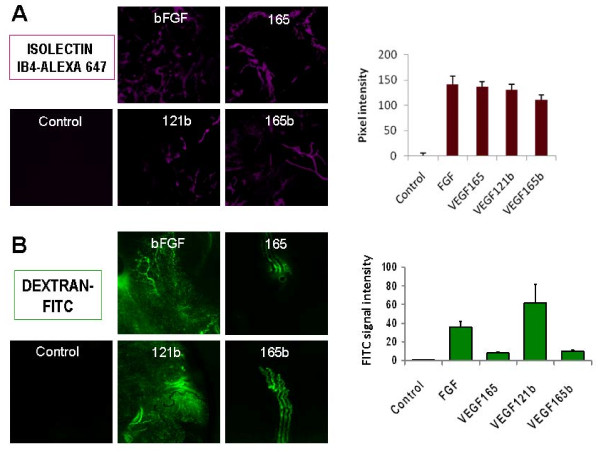
**VEGF_121/165_b isoforms induce vascularization *in vivo***. **A: **Angiogenesis analysis in Matrigel plugs with Alexa-647-labelled isolectin. No signal is found in controls, whereas Matrigel plugs carrying any of the VEGF_xxx_b isoforms show a strong signal, thus demonstrating angiogenesis *in vivo*. **B: **Analysis of Matrigels using FITC-labelled dextran. Control Matrigels do not show fluorescent signal; a large amount of FITC-labelled dextran is observed in VEGF_121_b- and bFGF-containing plugs, whereas VEGF_165_b- and VEGF_165_-containing plugs display a weaker signal.

Matrigel plug assays were done in order to decipher the effect of VEGF_121/165_b isoforms on endothelial cell function *in vivo*. Matrigel was mixed with either bFGF, or VEGF_165 _(from R&D), or VEGF_121_b(pp), or VEGF_165_b(pp). Blood vessels within the Matrigel plugs were identified by the presence of Alexa-647-labelled isolectin B4 (Figure 5A), which was injected systemically into the mice before the sacrifize. No signal from the Alexa-647-labelled lectin was observed in control Matrigels, whereas plugs carrying any of the VEGF_xxx_b isoforms showed a strong signal, thus demonstrating angiogenesis *in vivo *(Figure 5A). To analyze vascular permeability, FITC-labelled-dextran was injected in another set of mice with Matrigel plugs under similar experimental conditions (Figure 5B). Whereas almost no fluorescent signal was seen in control plugs, in Matrigels that were pre-loaded with bFGF or especially with VEGF_121_b, a dramatic increase in the fluorescent signal was found. Matrigel plugs carrying either VEGF_165 _or VEGF_165_b displayed a similar degree of fluorescence, which was approximately 10-fold higher than that of controls.

### VEGF_121/165_b overexpression stimulates tumor growth and angiogenesis *in vivo*

**Figure 6:**
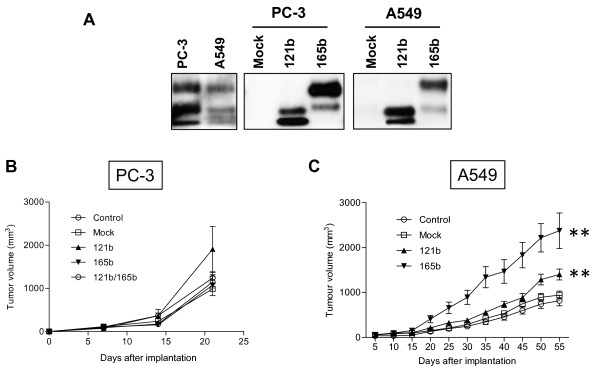
**VEGF_121/165_b overexpression accelerates tumorigenesis in A549 xenografts**. **A: **Western blot shows total endogenous VEGF expression in PC-3 and A549 cells, and VEGF_121/165_b-overexpression in the transfected cell pools. Two different exposure times were used to develop the blots: Long time (for endogenous VEGF protein levels in PC-3 and A549 cells) and short time (for transfected cells) exposure. Bands corresponding to glycosylated and non-glycosylated proteins can be observed. **B: **PC3 cells engineered to overexpress VEGF_121_b, VEGF_165_b, or both, injected subcutaneously into athymic mice. No statistical differences between groups are found, but tumors originated from injection of VEGF_121_b-overexpressing cells tend to be larger than those observed for the other groups. **C: **Tumors resulting from injection of A549 cells overexpressing VEGF_121/165_b isoforms are significantly larger (p < 0.01) than controls.

A cell line with high (PC-3) and another one with low (A549) endogenous total VEGF expression levels were selected for *in vivo *assays (Figure 6A). VEGF_121/165_b isoforms were overexpressed in both PC-3 and A549 xenograft models in order to assess the effect of these isoforms in tumor growth and angiogenesis. Western blot analyses showed a high expression of either VEGF_121_b or VEGF_165_b in cell pools that were selected with G418 over a period of 20 days (Figure 6A). No statistical differences in tumor growth were seen between controls and tumors overexpressing either VEGF_121_b, or VEGF_165_b, or a combination of both in PC3 xenografts (Figure 6B). Moreover, VEGF_121_b-overexpressing cells tended to form bigger tumors than the rest of the experimental conditions (Figure 6B). In the case of A549 xenografts, significant increases in tumor volumes were found when cells with either VEGF_121_b, or VEGF_165_b were injected, in comparison with controls (Figure 6C). Therefore, overexpression of the VEGF_121/165_b isoforms does not cause tumor shrinkage but tumor growth in these models.

**Figure 7:**
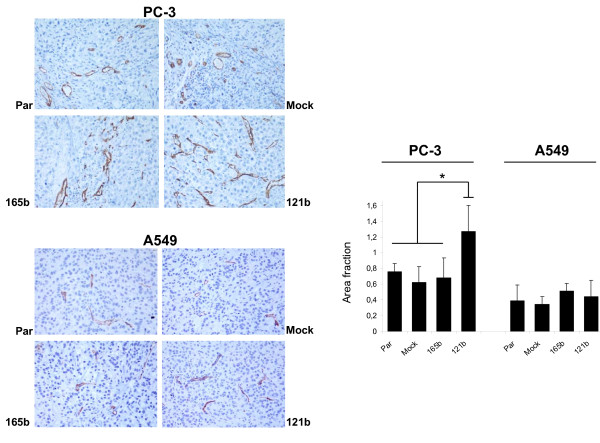
**Angiogenesis analysis in sections from xenografted tumors**. Immunostaining for CD-31 in A549 tumors revealed a poor vascularization and no differences between controls and VEGF_121/165_b-overexpressing tumors. In PC-3 tumors, a significant increase in angiogenesis was found for VEGF_121_b when compared with controls or VEGF_165_b-overexpressing tumors. Par: Parental cells.

Analysis of angiogenesis revealed that A549 tumors were less angiogenic than PC-3 tumors with no differences between controls and the experimental groups (Figure 7). In PC-3, VEGF_121_b-overexpressing tumors showed a significantly higher (p < 0.05) vascularization than the other experimental groups (Parental, Mock-transfected and VEGF_165_b). To analyze a possible mural recruitment to the vasculature, levels of PDGFRβ were analyzed by immunhostochemistry and image analysis. PDGFRs are expressed in pericytes and bone-marrow-derived cells that participate in blood vessel formation and coverage (22). Therefore, if VEGF_xxx_b isoforms alter the vascular wall, a decrease in PDGFRβ+ cells should be found in comparison with controls. However, no such decrease was observed in either PC-3 or A549 xenografted tumors (Additional File 2 Figure S2). We also quantified the number of apoptotic cells in these tumors, since an anti-angiogenic effect of the VEGF_xxx_b isoforms should be translated into an increase in apoptosis. In A549 xenografts, no changes were observed in the number of active caspase-3+ cells (Additional File 2 Figure S2). In PC-3 xenografted tumors, not an increase but a reduction in the number of apoptotic cells was observed when VEGF_xxx_b-overexpressing cells were injected, as compared to controls (Additional File 2 Figure S2). A significant reduction (~2-fold, p < 0.05) was found for VEGF_121_b, which is in keeping with the increased vascularization of these tumors.

### Expression of VEGF_xxx_b proteins and total VEGF-A in a set of normal breast and breast cancer samples

Previous studies had suggested that VEGF_xxx_b isoforms can be differentially expressed in normal vs. pathological conditions [13]. To study if this was the case for breast cancer, we used a TMA containing core biopsies from breast cancer patients and normal breasts. To this end, we used a well validated antibody that recognizes total VEGF-A (including all VEGF-A isoforms) (R&D systems) and the only available and validated anti-VEGF_xxx_b antibody that recognizes all the VEGF_xxx_b proteins (R&D systems) [18]. No available antibody specific for the "non-b" isoforms (VEGF_xxx_) is currently available.

**Figure 8:**
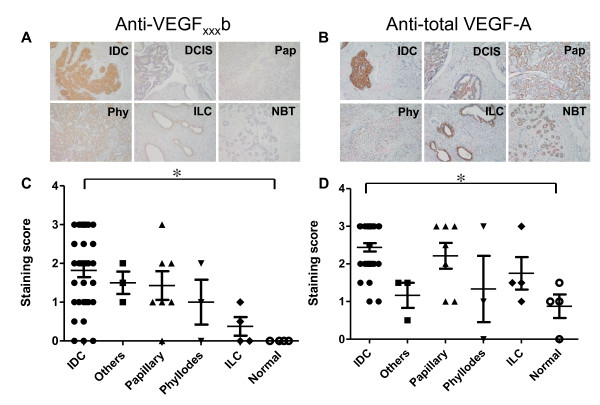
**VEGF_xxx_b protein expression is increased in human breast cancer samples in comparison to healthy mammary glands**. VEGF protein levels in breast cancer samples and healthy mammary glands were analyzed in a Tissue Microarray (TMA) by immunohistochemistry using an antibody raised specifically against exon 8b (A) and an anti-total VEGF-A antibody (B). The levels of both VEGF_xxx_b (C) and total VEGF-A (D) are higher in the breast cancer samples compared to normal breast tissues (NBT). Differences reach statistical significance when comparing normal breast glands with the Intraductal Carcinoma (IDC) subtype (p < 0.05) for both total VEGF and VEGF_xxx_b. Examples of staining include papillary carcinoma (Pap), phyllodes (Phy), infiltrating lobular carcinomas (ILC), and ductal carcinoma in situ (DCIS).

Figures 8A and 8B show representative images of malignant and normal breast tissues stained with the anti-VEGF_xxx_b and anti-total VEGF-A antibodies. Strong staining for VEGF_xxx_b could be seen in tumor cells of infiltrating ductal carcinoma (IDC) samples. Staining was also found for other types of tumors, including papillary carcinoma (Pap), phyllodes (Phy), infiltrating lobular carcinomas (ILC), and ductal carcinoma in situ (DCIS). No VEGF_xxx_b was detected in any of the normal breast tissue (NBT) samples. All tissues analyzed were positive for total-VEGF-A, including the normal breast epithelium. Semiquantification of the staining showed that both total VEGF-A and VEGF_xxx_b protein levels were significantly higher (p < 0.05) in IDC than in normal breast tissues (Figures 8C and 8D). A significant (p = 0.033) positive correlation index (r = 0.404) between VEGF_xxx_b and total-VEGF-A was found, thus indicating the degree of co-staining. Therefore, we conclude that VEGF_xxx_b levels are not reduced in malignant breast cancer but, on the contrary, tend to increase in the tumor samples and are significantly higher in infiltrating ductal carcinomas.

## Discussion

The importance of VEGF-A in normal and pathological angiogenesis has been widely explored and documented in the last decades [6]. VEGF-A has become a major target for cancer therapy in solid tumors, and VEGF-targeted drugs, such as bevacizumab or sunitinib are currently being used in patients [9,10]. The biology of VEGF-A spliced isoforms is, however, poorly understood yet. In particular, understanding how the different VEGF-A isoforms are generated through alternative splicing may be important to design more specific and effective molecular therapies.

In 2002, a new family of VEGF-A isoforms generated by alternative splicing was reported [14,19], which were denominated VEGF_xxx_b isoforms. These transcripts incorporate a different exon (called exon 8b, sequence SLTRKD) from the classical exon 8a (sequence CDKPRR) found in the angiogenic transcripts [19]. Inclusion of exon 8b was thought to endow VEGF_xxx_b isoforms with the capacity to bind VEGF-A receptors without strong downstream signalling activation [14]. Because of this property, the VEGF_xxx_b proteins were hypothesized to be antiangiogenic [14]. Although results have to be interpreted carefully, it was shown that overexpression of VEGF_165_b or VEGF_121_b in tumor cells xenotransplanted into nude mice results in growth inhibition [14,18,23-25]. Another interesting finding that was reported is that VEGF_xxx_b isoforms may be differentially expressed in pathological tissues compared to normal tissues [13,25,26]. Changes in the natural splicing would favor the amount of the VEGF_xxx _transcripts in some aberrant angiogenesis-linked diseases, at the expense of reducing the amount of the VEGF_xxx_b isoforms (mainly expressed in normal tissues).

Because of the great potential importance of these findings, we aimed in the present study to produce VEGF_121_b and VEGF_165_b recombinant proteins in the yeast *Pichia pastoris*, with the goal of testing their antiangiogenic/antitumor properties *in vitro *and *in vivo*. Generation of VEGF_121/165_b-overexpressing cancer cells using the PCDNA3.1 plasmid was also used for this purpose. The yeast expression system was chosen for several reasons. First, this system allows protein glycosylation, and purification with little secretion of yeasts-derived endogenous proteins that may contaminate the recombinant protein of interest. Another advantage is that protein production is easier, faster and cheaper than that generated in mammalian systems. In addition, we made sure with this expression system that no exon 8-containing VEGF-A contamination is present, since yeasts do not code for any form of VEGF. Producing VEGF-A in mammalian cells has the drawback of possible formation of exons 8 and 8b heterodimers. This may be of special importance, since VEGF-A is secreted and active in its dimeric form [6].

We have successfully produced recombinant VEGF_121_b and VEGF_165_b proteins that share similar structural characteristics to those of the classical VEGF_121 _and VEGF_165 _proteins: Ability to form dimers or multimers, and reactivity with commercial antibodies that were raised against exons 1 to 5 (common to all VEGF-A isoforms). In addition, both recombinant VEGF_121_b and VEGF_165_b proteins were immunoreactive with a validated antibody that recognizes exon 8b (from R&D [18]).

To test the functionality of these isoforms *in vitro*, HUVECs were treated with recombinant proteins produced in yeasts, VEGF_165_b produced in mammalian cells, or the "classical" VEGF_165 _angiogenic protein (as control). All treatments were carried out in serum-free media. We found that VEGF_121/165_b isoforms induced proliferation of HUVECs and phosphorylation of VEGFR2 and its downstream transducer ERK. The effect of VEGF_121/165_b isoforms was milder than that found for VEGF_165_: Proliferation of HUVECs was ~50% less stimulated with VEGF_121/165_b isoforms than that using recombinant VEGF_165_. However, the degree of ERK activation was similar for all the proteins tested, 10 min after stimulation. This intracellular effector was phosphorylated specifically by VEGFRs, as demonstrated by the inhibition of such process in the presence of the VEGFR1 and VEGFR2 tyrosine-kinase inhibitor GW654652.

Kawamura et al. [16] have demonstrated that VEGF_165_b displays unique functional characteristics. They showed VEGF_165_b to be a weak agonist of VEGFR2 and to induce its phosphorylation in HUVECs with a similar potency as VEGF_145_. Similarly to VEGF_121_, VEGF_165_b does not bind neuropilin-1 (NRP1) and failed to induce complexes between NRP1 and VEGFR2. VEGF_165_b promotes cell migration (in PAE cells transfected with VEGFR2), similar to VEGF_165_, VEGF_145_, and VEGF_121 _isoforms. Resembling the activity of VEGF_121 _and VEGF_145_, but unlike VEGF_165_, VEGF_165_b does not induce endothelial sprout. But unlike the other isoforms, VEGF_165_b was unable to phosphorylate the mouse VEGFR2 at the Y1052 position. Unfortunately, downstream signalling activation was not tested in this study [16]. Authors hypothesized that VEGF_165_b is not able to fully rotate the receptor's intracellular tail upon binding, thus blocking to some extent transphosphorylation [16]. In addition, Glass et al. [17] have shown that VEGF_165_b activates transiently VEGFR1 in order to elicit vascular permeability in the mesenteric vein. Collectively, these and our results suggest that VEGF_165_b may exert weak downstream signalling in endothelial cells. However, depending on the cellular context (for instance, the presence of NRP1 or the amount of VEGFR1 or VEGFR2), VEGF_165_b may block transphosphorylation to produce a non-angiogenic response.

To further validate the observation of VEGF_121/165_b angiogenic properties seen in classical *in vitro *assays, we conducted *in vivo *experiments with Growth Factor Reduced Matrigel, in which VEGF, among other angiogenic cytokines, have been significantly depleted. Addition of recombinant VEGF_121_b, or VEGF_165_b, or VEGF_165 _caused recruitment of blood vessels to the Matrigel compared to PBS-loaded controls, thus demonstrating an angiogenic effect. It is worth mentioning that VEGF_121_b-containing Matrigels were highly enriched in dextran-FITC signal, which seemed to be within and out of the vessels (similar to results found for bFGF). This suggests an increase in vascular permeability, in keeping with vascular permeability described for VEGF_121 _[27].

The ability to inhibit tumor growth *in vivo *was tested in our study by VEGF_121/165_b overexpression in xenograft models. We chose two different cancer cell lines: The low-VEGF-expressing lung adenocarcinoma A549 cell line (~1-5 pg/mL (28)) and PC-3, a prostate cancer cell line that secretes ~800 pg/mL; [29]. These cell types were chosen to ascertain possible differences in the VEGF_xxx_b behavior depending on the endogenous VEGF expression. Experiments were performed without any further exogenous VEGF stimulation, such as transfection of VEGF. In the PC3 xenografts, no statistical differences between controls and VEGF_121/165_b-expressing tumors were seen. However, a tendency of VEGF_121_b-overexpressing tumors to grow at a faster rate than the other groups was found. In agreement with these results, vascular density for VEGF_121_b-overexpressing PC-3 tumors was significantly higher than that observed for the rest of the groups, and apoptotic levels significantly lower. A549 xenografts grew slow and formed small tumors after subcutaneous implantation in nude mice, as previously described [30]. Strikingly, overexpression of VEGF_121_/_165_b in these cells resulted in a significant increase in tumor development over the controls, although no differences in vascular density were found between groups, which may be due to the poor angiogenic potential of these tumors.

These results are in conflict with previous reports showing that VEGF_165_b causes anti-tumor effects [14,15,23,24]. Our interpretation for these seemingly contradictory results is as follows: The *in vivo *models in which the VEGF_xxx_b isoforms have been proven to be efficacious in reducing tumor growth expressed high VEGF levels (either by the nature of the cells used or by transfection with VEGF-carrying plasmids). In these conditions, both VEGF_xxx _and VEGF_xxx_b proteins would compete equally for receptor binding. Because VEGF_xxx_b is only able to signal weakly, a reduced tumor volume will be observed when tumors overexpressing VEGF_xxx_b are compared to high VEGF-expressing tumors. However, in the case of tumors with low VEGF production, overexpression of VEGF_xxx_b would stimulate tumor growth to a certain extent. Thus, in CAKI cells, where endogenous total VEGF levels are ~900 pg/mL [23], when both parental and VEGF_165_b-overexpressing cells were injected into mice, no differences in tumor growth were found. Only when parental cells were transfected with VEGF_165_, a reduction in tumor volume was observed compared to VEGF_165_b-overexpressing tumors [23]. Similarly, significant differences in tumor volumes were only obtained in Mel57 (<600 pg/mL, [31]) and A375 (~600 pg/mL, [32]) melanoma xenograft models when VEGF_165_- and VEGF_165_b-overexpressing clones were compared [14,23]. On the contrary, in LS174t cells, which secrete high VEGF levels (~2000 pg/mL, [33]), overexpression of VEGF_165_b or VEGF_121_b [15], or administration of VEGF_165_b recombinant protein [24] led to tumor shrinkage compared to controls (parental cells with no further VEGF overexpression). CT71 Ewing sarcoma cell lines produce ~1200 pg/mL, [34]. When CT71 cells overexpressing VEGF_165_b were xenotransplanted into nude mice, a significant reduction in tumor volume was found when compared with untransfected CT71 xenografts [23].

It is possible then that therapies based on the administration of VEGF_xxx_b proteins are effective in tumors with high endogenous VEGF expression. However, the use of this therapy in tumors with low VEGF levels, which likely rely on other angiogenic factors for their growth (such as bFGF, IL-8, etc.) may worsen the evolution of the tumor. Therefore, therapy with VEGF_xxx_b casts some doubts about its possible use in unselected patients. Caution must be taken in defining which possible patient will benefit from VEGF_xxx_b-based therapies. Stratifying the patients based on the amount of VEGF production may be critical for future possible clinical trials.

But before translating this type of therapy, many aspects of the VEGF_xxx_b biology should be clarified. For instance, it is quite surprising that VEGF_121_b, although found to inhibit endothelial cell migration, was cytoprotective for endothelial cells (in serum starvation experiments), in a similar way as VEGF_165 _[15]. Such cytoprotection involved activation of VEGF receptors and downstream signalling [15]. Even more surprising is the fact that in some xenograft models (for instance using CAKI cells), co-overexpression of VEGF_165 _and VEGF_165_b results in tumors that are smaller in size than those generated by overexpression of just VEGF_165_b [23]. Other issues, such as whether or not VEGF_xxx_b proteins are able to heterodimerize with members of the VEGF_xxx _angiogenic family, should be clarified as well.

We have also addressed in this study the issue of whether the VEGF_xxx_b isoforms may be differentially expressed in malignant tissues (human breast cancer) compared to healthy tissues (normal mammary gland). For our experiments we used immunohistochemistry with validated antibodies specific for all VEGF_xxx_b isoforms and antibodies that recognize all VEGF transcripts. Our results revealed that both total VEGF and VEGF_xxx_b levels tended to increase in breast cancer samples (n = 50) compared to normal breast tissues (n = 8). This increase was statistically significant for intraductal carcinomas (IDC). Expression of both total VEGF and VEGF_xxx_b were significantly correlated, thus suggesting that both families of VEGF (VEGF_xxx _and VEGF_xxx_b) follow a similar pattern of expression.

Previous studies have analyzed, in a limited number of samples, expression of the VEGF_xxx_b isoforms. Whereas total VEGF mRNA levels were found significantly up-regulated in colon carcinoma samples (n = 6) compared to controls, no changes were observed for VEGF_xxx_b mRNA levels [18]. This result shows that the increase in total VEGF is due to the VEGF_xxx _angiogenic isoforms [18]. A similar result was found analyzing protein levels by isoform-specific ELISAs [18]. RT-PCR analysis showed that VEGF_165_b was present in 17 of 18 normal kidney samples, but only in 4 of 18 matched malignant tissues [19]. Immunohistochemical analysis of 19 melanoma samples (9 metastatic and 10 nonmetastatic) using the VEGF_xxx_b specific antibody, found a decrease in VEGF_xxx_b expression in the neoplastic tissue (especially in metastasis) compared to the normal skin [25].

The lack of appropriate antibodies specific for each of the VEGF_xxx _and VEGF_xxx_b proteins hinders the fine characterization of the pattern of expression in malignant and normal tissues. Future studies using larger number of samples, and quantitative real time RT-PCR and immunohistochemical analyses would be needed to assess whether VEGF_xxx_b expression could be used as a cancer biomarker.

## Conclusions

Our results demonstrate that VEGF_121/165_b are not anti-angiogenic, but weakly angiogenic isoforms of VEGF-A that may foster tumor growth and angiogenesis *in vivo*. We also conclude that VEGF_xxx_b isoforms (as well as total VEGF levels) are up-regulated in breast cancer in comparison with non malignant breast tissues.

## Competing interests

The authors declare that they have no competing interests.

## Authors' contributions

RC participated in the design of the study and undertook the vast majority of the experimentation, analysis of the data, and writing of the manuscript. LL, ML and JA conducted western blots and in vivo experiments. EM, RM, and JH participated in the production and purification of VEGF_121/165_b in *Pichia pastoris*. RP and LM participated in the study design and manuscript editing. AC contributed in the study design, monitoring of the experimentation and writing the paper. All authors read and approved the manuscript.

## Supplementary Material

Additional file 1 **Figure S1. Deglycosylation of VEGF_121_b and VEGF_165_b proteins produced in *Pichia pastoris *in reducing and non-reducing conditions**. Treatment of both proteins with Endoglycosidase F1 produces an electrophoretic shift. VEGF_121_b and VEGF_165_b are glycosylated and the molecular weights of both glycosylated and deglycosylated proteins are compatible with those described for VEGF_121 _and VEGF_165_. In non-reducing conditions, glycosylated dimers of VEGF_121_b and VEGF_165_b display 41 and 28 KDa molecular weight, respectively, as previously described. After deglycosylation, molecular weight of dimers is decreased by around 10 KDa, similar to mammalian deglycosylation of VEGF. Under reducing conditions (where monomers are observed) both VEGF_121_b and VEGF_165_b glycosylated bands disappear upon Endoglycosidase treatment. The 30 KDa band that is observed in reducing conditions corresponds to the Endoglycosidase F1 present in the mixture reaction. Click here for file

Additional file 2 **Figure S2. Analysis of active caspase-3 and PDGFRβ protein expression in sections from xenografted tumors**. Immunostaining for active caspase-3 in A549 tumors shows no changes in apoptotic rates in VEGF_121/165_b-overexpressing tumors, as compared with controls. In PC-3 tumors, a significant reduction in apoptosis is observed for VEGF_121_b when compared to controls. Immunohistochemistry for PDGFRβ+ cells shows no differences in expression between controls and VEGF_121/165_b-overexpressing groups, in either PC-3 or A549 xenografted tumors. Click here for file
