# Supplementary material for: VEGF121b and VEGF165b are weakly angiogenic isoforms of VEGF-A
Source: Mol Cancer. 2010 Dec 31;9:320. doi: 10.1186/1476-4598-9-320 (PMC3022671; doi:10.1186/1476-4598-9-320)
Supplement: Additional file 1 — Figure S1. Deglycosylation of VEGF121b and VEGF165b proteins produced in Pichia pastoris in reducing and non-reducing conditions. Treatment of both proteins with Endoglycosidase F1 produces an electrophoretic shift. VEGF121b and VEGF165b are glycosylated and the molecular weights of both glycosylated and deglycosylated proteins are compatible with those described for VEGF121 and VEGF165. In non-reducing conditions, glycosylated dimers of VEGF121b and VEGF165b display 41 and 28 KDa molecular weight, respectively, as previously described. After deglycosylation, molecular weight of dimers is decreased by around 10 KDa, similar to mammalian deglycosylation of VEGF. Under reducing conditions (where monomers are observed) both VEGF121b and VEGF165b glycosylated bands disappear upon Endoglycosidase treatment. The 30 KDa band that is observed in reducing conditions corresponds to the Endoglycosidase F1 present in the mixture reaction. [file 1476-4598-9-320-S1.PPT]

## Slide 1
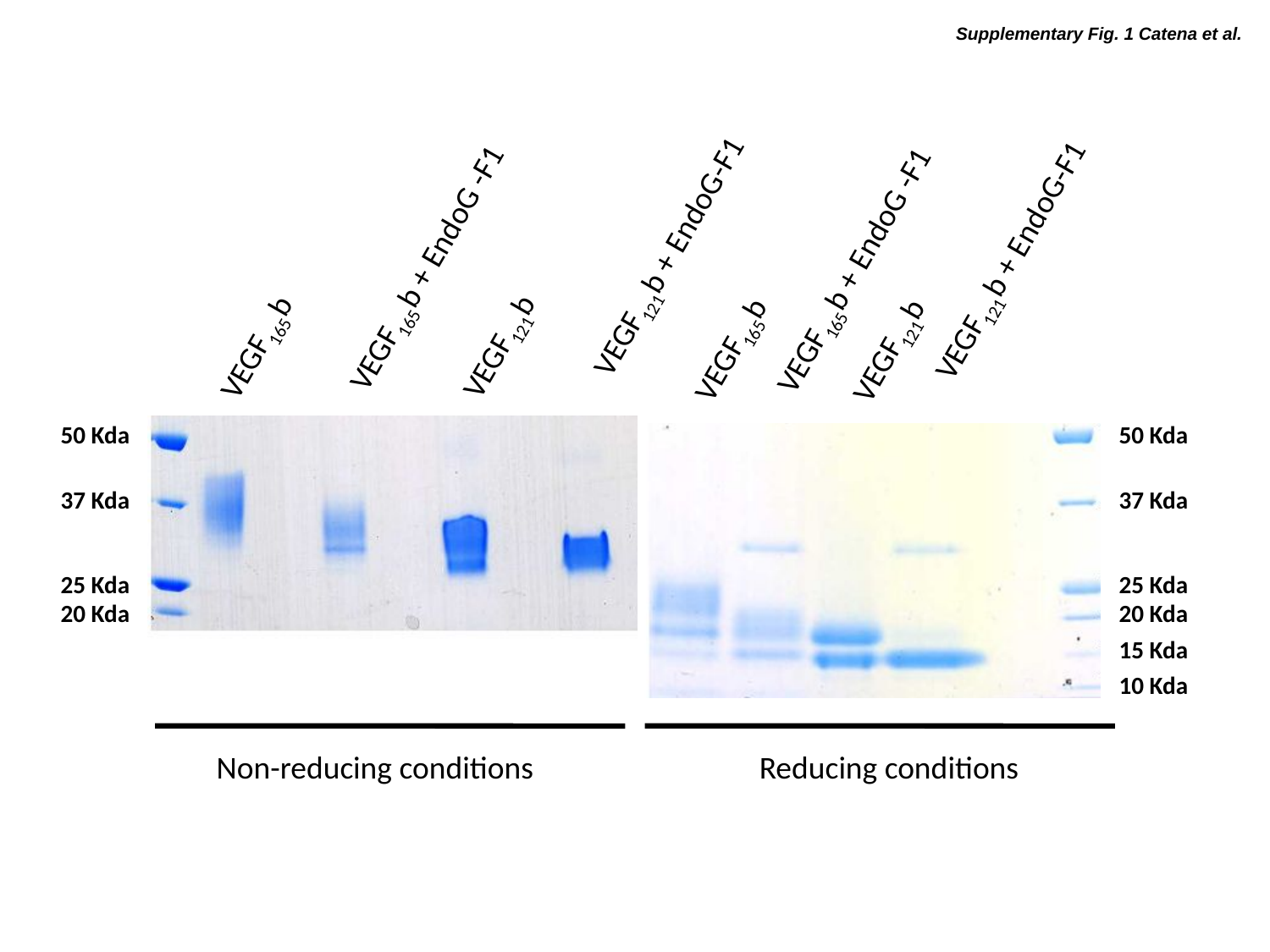

Supplementary Fig. 1 Catena et al.
VEGF121b + EndoG-F1
VEGF121b + EndoG-F1
VEGF165b + EndoG -F1
VEGF165b + EndoG -F1
VEGF121b
VEGF165b
VEGF165b
VEGF121b
50 Kda
50 Kda
37 Kda
37 Kda
25 Kda
25 Kda
20 Kda
20 Kda
15 Kda
10 Kda
Non-reducing conditions
Reducing conditions
