# Supplementary material for: VEGF121b and VEGF165b are weakly angiogenic isoforms of VEGF-A
Source: Mol Cancer. 2010 Dec 31;9:320. doi: 10.1186/1476-4598-9-320 (PMC3022671; doi:10.1186/1476-4598-9-320)
Supplement: Additional file 2 — Figure S2. Analysis of active caspase-3 and PDGFRβ protein expression in sections from xenografted tumors. Immunostaining for active caspase-3 in A549 tumors shows no changes in apoptotic rates in VEGF121/165b-overexpressing tumors, as compared with controls. In PC-3 tumors, a significant reduction in apoptosis is observed for VEGF121b when compared to controls. Immunohistochemistry for PDGFRβ+ cells shows no differences in expression between controls and VEGF121/165b-overexpressing groups, in either PC-3 or A549 xenografted tumors. [file 1476-4598-9-320-S2.PPT]

## Slide 1
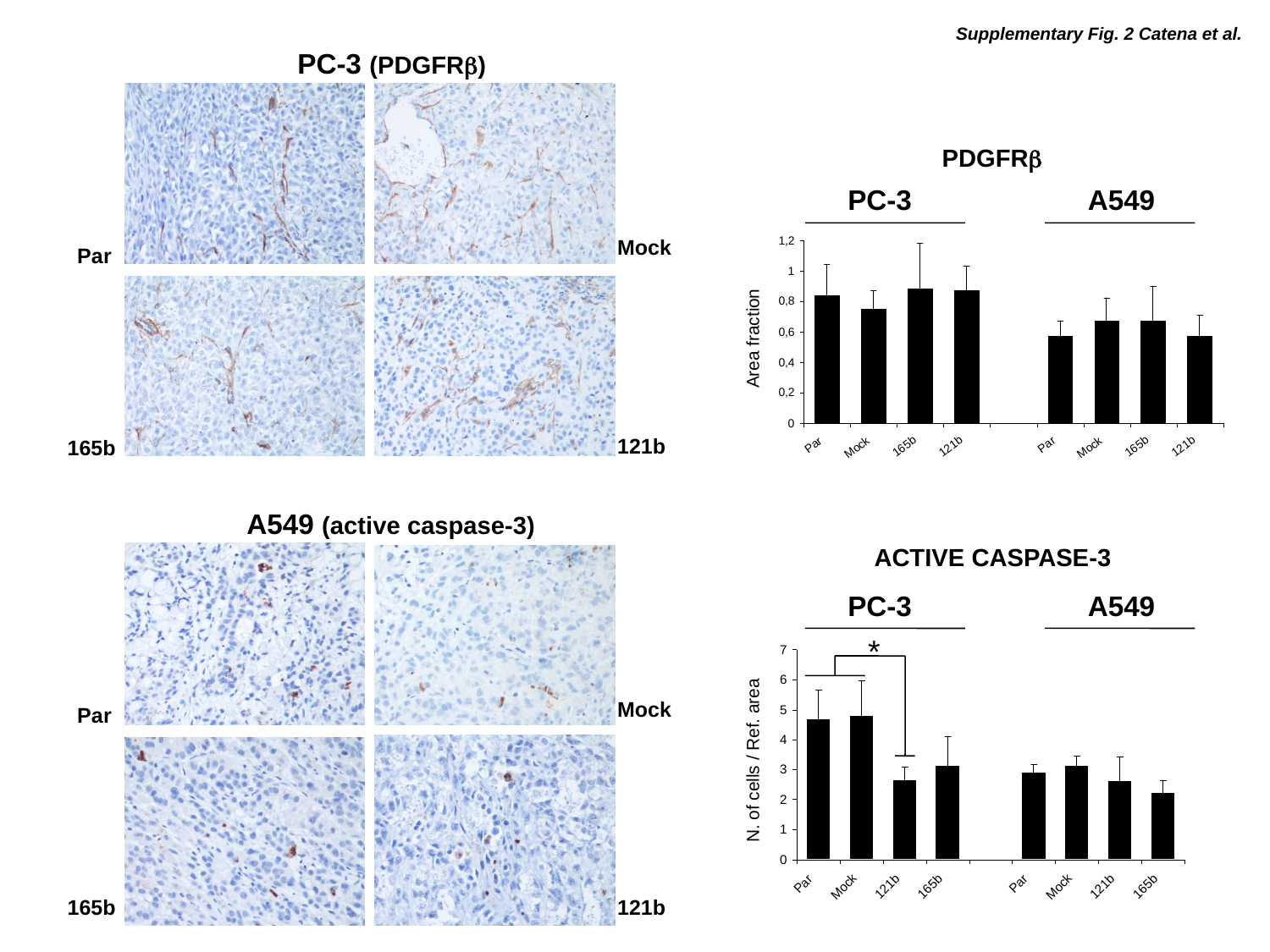

Supplementary Fig. 2 Catena et al.
PC-3 (PDGFRβ)
Mock
Par
121b
165b
A549 (active caspase-3)
Mock
Par
165b
121b
PDGFRβ
PC-3
A549
Area fraction
ACTIVE CASPASE-3
PC-3
A549
*
N. of cells / Ref. area
